# Experimental transmission of Zika virus by mosquitoes from central Europe 

**DOI:** 10.2807/1560-7917.ES.2017.22.2.30437

**Published:** 2017-01-12

**Authors:** Anna Heitmann, Stephanie Jansen, Renke Lühken, Mayke Leggewie, Marlis Badusche, Björn Pluskota, Norbert Becker, Olli Vapalahti, Jonas Schmidt-Chanasit, Egbert Tannich

**Affiliations:** 1 Bernhard Nocht Institute for Tropical Medicine, Hamburg, Germany; 2 These authors contributed equally to this work; 3 German Centre for Infection Research (DZIF), partner site Hamburg-Luebeck-Borstel, Hamburg, Germany; 4 Institute for Dipterology (IfD), Speyer, Germany; 5 University of Heidelberg, Heidelberg, Germany; 6 University of Helsinki and Helsinki University Hospital, Helsinki, Finland

**Keywords:** Zika virus, Culex species, Aedes albopictus, transmission rate

## Abstract

Mosquitoes collected in Germany in 2016, including *Culex pipiens pipiens* biotype *pipiens*, *Culex torrentium* and *Aedes albopictus*, as well as *Culex pipiens pipiens* biotype *molestus* (in colony since 2011) were experimentally infected with Zika virus (ZIKV) at 18 °C or 27 °C. None of the *Culex* taxa showed vector competence for ZIKV. In contrast, *Aedes albopictus* were susceptible for ZIKV but only at 27 °C, with transmission rates similar to an *Aedes aegypti* laboratory colony tested in parallel.

In 2015, Zika virus (ZIKV) emerged in Columbia and Brazil and spread rapidly across the American continent and the Caribbean, causing an epidemic with notable numbers of associated clinical cases of microcephaly and Guillain–Barré syndrome [1]. Mosquitoes of the species *Aedes aegypti* and *Ae. albopictus* are considered the primary and secondary vectors of ZIKV [2]. However, with transmission rates below 50%, their vector competence for ZIKV in the laboratory is low [3]. The question therefore remains whether other common mosquito species such as *Culex* spp. play a role in the transmission cycle of ZIKV. The few studies performed so far have provided inconclusive results and suggested that at least *Culex quinquefasciatus* might be able to transmit ZIKV [4-9]. In addition, for an assessment of the risk of possible spread to regions with temperate climate such as central Europe, information is lacking on ZIKV vector competence of mosquitoes under reduced temperature conditions (< 20°C). 

This study aimed to evaluate the vector competence of central European mosquito species for ZIKV. Therefore, German populations of *Culex pipiens pipiens* biotype *pipiens* (*Cx. p. pipiens*), *Culex pipiens pipiens* biotype *molestus* (*Cx. p. molestus*), *Culex torrentium* and *Ae. albopictus* (*Ae. albopictus*, GER) were experimentally infected with ZIKV, using *Ae. aegypti* and an Italian *Ae. albopictus* (*Ae. albopictus*, ITA) as positive controls. 

## Experimental infection of mosquitoes

Two long-established laboratory strains (*Ae. aegypti* (Bayer company) and *Cx. p. molestus* (in colony since 2011, collected in Heidelberg, Germany)) and four species collected in summer 2016 (*Cx. p. pipiens* F0 (collected in Hamburg, Germany), *Culex torrentium* F0 (collected in Hamburg, Germany), *Ae. albopictus* F7 (collected in Freiburg, Germany) and *Ae. albopictus* F7 (collected in Calabria, Italy)) were analysed and maintained as previously described [10,11]. All colonies tested negative in pan-flavivirus PCRs [12].

Between 150 and 200 female mosquitoes 4–14 days-old were starved for 24 h before application of infectious blood meals containing ZIKV (strain ZIKV_FB-GWUH-2016, GenBank KU870645, fifth passage) [13] at a final concentration of 10^7^ plaque-forming units (PFU)/mL. Artificial feeding was performed using a Hemotek Feeder (*Aedes* spp.) or by cotton sticks (*Culex* spp.). Engorged females were incubated at 80% humidity at either 18 °C or 27 °C. Analyses for ZIKV were done 14 and 21 days post infection (dpi) for approximately 35 randomly selected females and twice the number for *Ae. aegypti* at 27°C. For salivation, mosquitoes were anaesthetised and the proboscises were inserted into cropped 10 µL filter tips containing 10 µL phosphate-buffered saline (PBS). After 30 min, tips were removed and saliva-containing PBS was analysed for the presence of infectious virus particles by measuring its cytopathic effect (CPE) on Vero cells within the following 8 days. ZIKV in the supernatant of cytopathic cells was confirmed by qRT-PCR using Real Star Zika Virus RT-PCR Kit (Altona diagnostics, Hamburg, Germany). In addition, bodies of all challenged mosquitoes, excluding legs and wings, were analysed for ZIKV RNA by qRT-PCR.

## Results

At 14 or 21 dpi, ZIKV RNA was detected in the bodies of all challenged mosquito taxa, with infection rates ranging between 3 and 72% in the species–temperature combinations with ZIKV-positive bodies. Infection rates and virus titres were substantially higher in *Aedes* species, with viral RNA copies ranging from 10^2^ to 10^4^ in *Culex* spp. and from 10^4^ to 10^9^ in *Aedes* spp. (Table).

**Table:** Susceptibility and transmission rates of mosquitoes experimentally infected with Zika virus (n = 856)

		14 days post infection			21 days post infection		
Mosquito taxa	T in °C	IR^a^ (%)	Mean (SD) log10 RNA copies/specimen^b^	TR^c^ (%)	IR^a^ (%)	Mean (SD) log10 RNA copies/specimen	TR (%)
*Aedes aegypti*	18	17/31 (55)	4.70 (0.86)	0/17	18/33 (55)	4.33 (0.63)	0/18
	27	31/63 (49)	8.69 (1.60)	14/31 (45)	36/50 (72)	6.82 (1.75)	11/36 (31)
*Aedes albopictus*, ITA	18	19/30 (63)	4.05 (0.59)	0/19	14/39 (36)	5.52 (0.87)	0/14
	27	22/31 (71)	6.34 (2.14)	4/22 (18)	15/29 (52)	7.41 (2.22)	2/15 (13)
*Aedes albopictus*, GER	18	4/32 (13)	6.22 (1.25)	0/4	11/32 (34)	6.36 (1.39)	0/11
	27	20/31 (65)	6.78 (2.41)	4/20 (20)	18/34 (53)	8.61 (1.82)	6/18 (33)
*Culex p. molestus*	18	12/41 (29)	3.40 (0.38)	0/12	2/32 (6)	2.48 (0.29)	0/2
	27	7/29 (24)	3.73 (0.38)	0/7	12/38 (32)	4.02 (0.44)	0/12
*Culex p. pipiens*	18	16/34 (47)	3.38 (0.40)	0/16	3/32 (9)	3.88 (0.43)	0/3
	27	3/37 (8)	3.13 (0.45)	0/3	0/35 (0)	NA^d^	NA^d^
*Culex torrentium*	18	11/35 (31)	3.15 (0.47)	0/11	1/38 (3)	3.31 (NA)	0/1
	27	4/36 (11)	3.80 (1.79)	0/4	0/34 (0)	NA^d^	NA^d^

GER: from Germany; IR: infection rate; ITA: from Italy; NA: not available; SD: standard deviation; T: temperature; TR: transmission rate.

^a^ Infection rate: number of ZIKV-positive mosquito bodies per number of fed females.

^b^ RNA copies were averaged over all ZIKV-positive mosquito bodies excluding the zeros of ZIKV-negative mosquito bodies.

^c^ Transmission rate: number of mosquitoes with ZIKV-positive saliva per number of ZIKV-positive mosquito bodies.

^d^ Not available: Mean viral RNA copies and transmission rate could not be calculated for the species–temperature combinations with no ZIKV-positive bodies.

Virus load was generally higher at elevated incubation temperature (27 °C vs 18 °C). However, transmission of infectious virus particles as measured by CPE of Vero cells incubated with mosquito saliva was not detected in any of the *Culex* taxa. In contrast, saliva was positive for infectious virus particles in all *Aedes* species, but only at 27 °C incubation temperature. Interestingly, transmission rates at 21 dpi were similar in *Ae. aegypti* and *Ae. albopictus* from Germany but were substantially lower in *Ae. albopictus* from southern Italy (30% vs 13%).

## Discussion

*Culex* species from central Europe are known as established vectors, able to transmit numerous viruses including West Nile, Sindbis and Usutu virus [14,15]. The results presented here indicate that the three most common *Culex* taxa in central Europe (*Cx. p. pipiens*, *Cx. p. molestus* and *Cx. torrentium*) do not have vector competence for ZIKV. This is in agreement with results from other parts of the world including Italy [4-7,9], which all showed a low degree of compentence of the *Cx. pipiens* complex for ZIKV transmission.

The invasive mosquito *Ae. albopictus* is established in large parts around the Mediterranean Sea and is considered to be the main vector in Europe for autochthonous human infections with chikungunya and dengue virus [16]. *Aedes albopictus* are regularly introduced into Germany as accidental cargo via road traffic from southern Europe [17]. In the winter 2015/16, successful overwintering of the species was observed for the first time in southern Germany [18]. The results presented here indicate that specimens of this overwintering population have considerable susceptibility to ZIKV, although only at elevated temperature of 27 °C. Moreover, the transmission rate in this overwintering population was substantially higher than in *Ae. albopictus* from the Calabrian region in southern Italy. Whether the difference in virus susceptibility between German and Italian *Ae. albopictus* populations is due to an ongoing process of adaptation to a new environment or to experimental conditions remains to be determined. Nevertheless, the susceptibility of European *Ae. albopictus* to ZIKV demonstrates the risk of arbovirus transmission associated with the establishment and ongoing spread of this invasive mosquito species in Europe. Of note, none of the tested *Aedes* populations were susceptible to ZIKV at 18 °C, which may limit the spread of ZIKV in central Europe to short summer periods with high temperatures. However, for a comprehensive risk assessment of ZIKV transmission in central Europe, further infection studies are needed at intermediate temperatures (e.g. 21 °C and 24 °C) as well as with other common *Aedes* species such as *Ae. vexans* or the newly established *Ae. japonicus* [19].
